# A resource for assessing dynamic binary choices in the adult brain using EEG and mouse-tracking

**DOI:** 10.1038/s41597-022-01538-5

**Published:** 2022-07-16

**Authors:** Kun Chen, Ruien Wang, Jiamin Huang, Fei Gao, Zhen Yuan, Yanyan Qi, Haiyan Wu

**Affiliations:** 1 Centre for Cognitive and Brain Sciences, University of Macau, Taipa, Macau SAR China; 2 Department of Psychology, Faculty of Social Sciences, University of Macau, Taipa, Macau SAR China; 3 Department of Sociology, Faculty of Social Sciences, University of Macau, Taipa, Macau SAR China; 4 Faculty of Arts and Humanities, University of Macau, Taipa, Macau SAR China; 5 Faculty of Health Sciences, University of Macau, Taipa, Macau SAR China; 6 Department of Psychology, School of Education, Zhengzhou University, Zhengzhou, China

**Keywords:** Decision, Motor cortex

## Abstract

We present a dataset combining high-density Electroencephalography (HD-EEG, 128-channels) and mouse-tracking intended as a resource for examining the dynamic decision process of semantics and preference choices in the human brain. The dataset includes resting-state and task-related (food preference choices and semantic judgments) EEG acquired from 31 individuals (ages: 18–33). Along with the dataset, we also provided the preliminary microstate analysis of resting-state EEG and the ERPs, topomap, and time-frequency maps of the task-related EEG. We believe that the simultaneous mouse-tracking and EEG recording would crack the core components of binary choices and further index the temporal dynamics of decision making and response hesitation. This publicly available dataset could support the development of neural signal processing methods in motor EEG, thus advancing research in both the decision neuroscience and brain-computer interface (BCI) applications.

## Abstract (summary)

**Table:** 

Measurement(s)	brain measurement (resting/task) • mouse trajectory; binary decision-making
Technology Type(s)	high-density EEG • Experiment

## Background & Summary

Mouse-tracking is an emerging approach for a real-time recording of motion trajectory by using the computer-based pointing device^1^, and its use has been continuously increasing in psychology and neuroscience studies. Mouse-tracking could provide both spatial and temporal features that allow the investigation of cognitive processing and could work as a novel measure to improve individual detection in experimental settings. For example, Sullivan *et al*. used mouse-tracking in a food choice task and found that the tastiness processing is 195 ms earlier than healthfulness processing^2^.

Most of the existing neuroimaging studies combined with mouse-tracking were functional magnetic resonance imaging (fMRI) studies^3–5^. These data were not publicly available, and the relatively low temporal resolution of fMRI limits its use in tracking fast temporal neural dynamics underlying the decision and execution of the choices. Alternatively, EEG is a non-invasive electrophysiological technique with high temporal resolution, which could track the dynamics of decisions at a millisecond time scale. Importantly, the openness of EEG/Magnetoencephalography (MEG) datasets during task-free or task-related paradigms^6–8^ is increasing, which merits advances in automatic data processing and research reproducibility. For example, the open dataset from Human Connectome Project (HCP), which provides both resting-state and task-related high-density EEG data, has been cited over 1000 times^9^, indicating mass attention received. However, the combination of mouse-tracking and EEG during different tasks is still missing.

One obvious obstacle to existing EEG mouse-tracking approach is the hand movement artifacts. Previous neural decoding studies and brain-computer interface (BCI) studies have applied EEG in mouse control, which could provide participants (health control or patients with motor disabilities) with cursor movement and target selection^10–12^. Although mouse control is a crucial component during EEG-based BCI, little is known about the mouse- or decision-related motor effect for EEG signals in humans. Most studies that used raw EEG data rarely provided artifact removal details or datasets with a synchronized recording of EEG and mouse-tracking.

Here, we provide the first open-access HD-EEG (128-channels) dataset recorded during resting-state and three binary choice tasks (food preference, word choice and image choice). During the mouse-tracking along with EEG, both task-related motor responses and mental processing could be reflected in the behavioral and brain patterns. These open data can be used for classifying motor noise and EEG signals related to decision processes. We have preliminarily shown the EEG patterns when people make decisions in image/text-based semantic judgment task, food preference task, and resting-state based on this dataset. This data from different decision tasks can further provide brain data in computer cursor controlling or identifying temporal dynamic patterns in responses or choices.

We anticipate that the current work could encourage decision neuroscience, cognitive neuroscience, and biomedical engineering researchers to reuse the dataset for brain activation pattern analyses, artificial removal, and neural decoding.

## Methods

### Participants and task overview

Thirty-one college students (18–33 years old, average: 20.68 years old; 14 males) participated in the present experiment. None of the participants reported any neurological or psychiatric history. All participants were right-handed and had normal or corrected-to-normal vision. Each participant voluntarily enrolled in and signed an informed consent form prior to the experiments and got the monetary compensation of approximately MOP 60 for one experimental session. The anonymous participants can only be identified by the tag from “sub-01” through “sub-31”. This study was performed in strict accordance with the Guidelines for Research Ethics of the University of Macau. The Institutional Review Board of the University has approved all procedures.

Text descriptions of task overview were shown in Table 1.

**Table 1:** An overview of the four sessions.

Session	Description
Resting-state	Measures EEG activity during rest.
Food choice task	A food preference task based on mouse-tracking. Measures the dynamic process and corresponding EEG signals of food preferences.
Image choice task	A semantic classification task for images based on mouse-tracking. Measures the dynamic semantic process and corresponding EEG signals of binary decisions.
Word choice task	A semantic classification task for written words based on mouse-tracking. Measures the dynamic semantic process and corresponding EEG signals of binary decisions.

### Experimental procedures

Participants were instructed to sit in an adjustable chair, whose eyes were approximately 60 cm away from the monitor (Dell, resolution: 1,920 × 1,080 pixels, vertical refresh rate: 60 Hz), see Fig. 3a. They were then informed that they would perform simple decision making tasks. All three tasks were divided into three blocks, interleaved with two breaks when the experimenter could check the impedance of the electrodes and supplement saline if necessary.

Stimulus presentation and manual response measurement were controlled by PsychoPy^13^ Standalone (2020.2.3), and the EGI PyNetstaion module was used to connect PsychoPy and EGI Netstation. All stimuli were presented on the screen against a black background (RGB: 0,0,0). A white cross subtended 1.0 × 1.0° worked as a fixation at the center of the screen. There was a grey rectangle (RGB: 169, 169, 169) with the white-font “start” subtended 4.0 × 2.2° at the start of each trial, showing up at the bottom center of the screen. Two target options (images or words) subtended 10.6 × 8.0°, appeared at the screen’s top left and top right side, respectively. After clicking on one of the targets, a blue frame (RGB: 0, 255, 0) will appear on the selected target, subtended 10.7 × 8.1°.

#### Session 1: Resting-state

The resting-state session consisted of two blocks, with 50 trials in each block. In each trial, participants were asked to look at the fixation cross at the center of the screen for 7 s, followed by a blank lasting for 1 s. There was a 2-min break between the two blocks.

#### Session 2: Food choice task

Three hundred and twenty pictures depicting various types of food were selected from Food-Pics Extended^14^ for both the pre-rating and food choice tasks. Firstly, participants were required to rate the target food from three aspects, which were presented randomly.

The first rating task aims to examine participants’ preferences in food choices. Participants needed to rate the taste of the target food presented at the center of the screen by a five-point Likert scale (“How do you like this food?” One denotes “extremely unsavory” while five denotes “extremely delicious”). The second rating relates to participants’ perception of food healthiness. Participants were asked to judge whether the target food was healthy or not (“How healthy do you think this food is?” One denotes “extremely unhealthy” while five denotes “extremely healthy”). In the third rating, participants were asked to judge how much they wanted to eat the target food after the experiment (“How much do you want to eat this food after the experiment?” One denotes “I don’t want it at all” while five denotes “I desperately want it”).

**Fig. 1:**
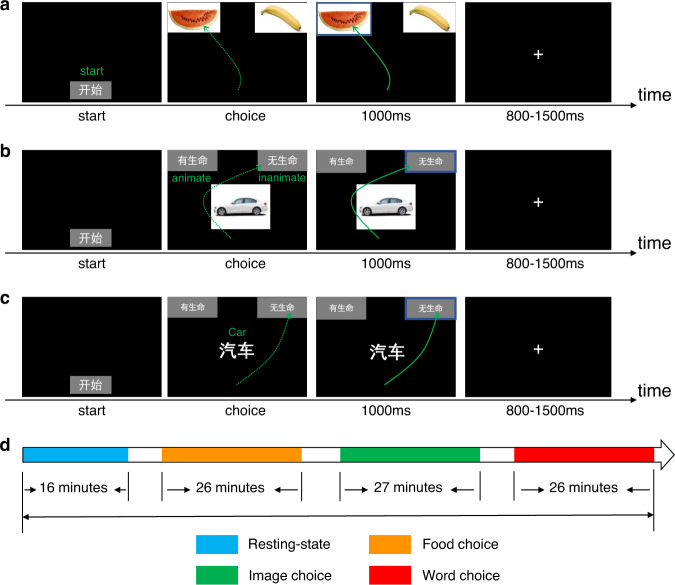
Procedures and timeline of the four sessions. (**a**) Procedure of the food choice task. (**b**) Procedure of the semantic category task with image modality. (**c**) Procedure of the semantic category with text modality. (**d**) Timeline of the four different sessions.

In the formal food choice task, as shown in Fig. 1a, each trial began with a blank with the word “start” at the bottom center of the screen. The participant could click the start box once they were ready. Two pictures of target food appeared in the upper left and upper right corner of the screen. The participants were asked to choose the preferred one by continuously moving the mouse to their favorite food box and clicking it. Upon click, the selected picture would be highlighted in blue for 1000 ms. After the pictures disappeared, a fixation would show up for 800 to 1500 ms (mean = 1150 ms). There were 160 pairs of pictures in the formal task, with each pair appearing twice. This session took about 26 minutes. There were two 2-min breaks in this task.

#### Session 3: Image choice task

Eighty images of animate objects and 80 inanimate objects were collected and designed by ourselves. Each image appeared twice in a pseudo-randomized order. Each trial began with the “start” button at the bottom center of the screen, as shown in Fig. 1b. Once started, the target object image would appear at the center. Participants need to determine whether the object is an animate or inanimate thing by clicking on the corresponding option in the upper left or upper right corner of the screen with a mouse. Upon click, the selected option would be highlighted in blue and lasted for 1000 ms. A fixation cross would subsequently show up and last for 800–1500 ms randomly. There were 320 trials in total. The positions for the “animate” and “inanimate” options were counterbalanced across all trials. It took participants around 27 minutes to complete this session. There were two 2-min breaks in this task.

#### Session 4: Word choice task

The procedure of the word choice task was almost identical with the image choice task, as shown in Fig. 1c. All the pictures from session three were replaced by their corresponding Chinese written words. To avoid ambiguity, two raters of Chinese linguistics background were invited to name the pictures and reached an agreement upon the 80 animate and 80 inanimate nouns. Two word types were also matched regarding word frequency [t(80) = 1.241, p = 0.216, word frequency data retrieved from the chinese corpus (http://corpus.zhonghuayuwen.org)] and number of strokes [t(80) = 0.749, p = 0.455]. This session took around 26 minutes. There were two 2-min breaks in this task.

Although the time to finish all of the tasks is long, we attempted to give participants enough break time during the task and between the tasks. Meanwhile, the saltwater during the break time can keep the impedance of the electrodes low, which provides sufficient guarantees to ensure high quality EEG data collection.

## Data Records

All data are publicly accessible (https://openneuro.org/datasets/ds003766)^15^ in the brain imaging data structure (BIDS)^16^ format under the OpenNeuro platform.

### EEG data collection

As shown in Fig. 3a,b, EEG data were acquired using a 128-channel cap based on the standard 10/20 System with Electrical Geodesics Inc (EGI, Eugene, Oregon) system. The layout of EEG electrodes was presented in Fig. 3b. During recording, the sampling rate was 1000 Hz, and the E129 (Cz) electrode was used as reference. Electrode impedances were kept below 50k Ω for each electrode during the experiment. The raw EEG data was exported to metafile format (.mff) files on the Mac OS.

### EEG data organization

**Fig. 2:**
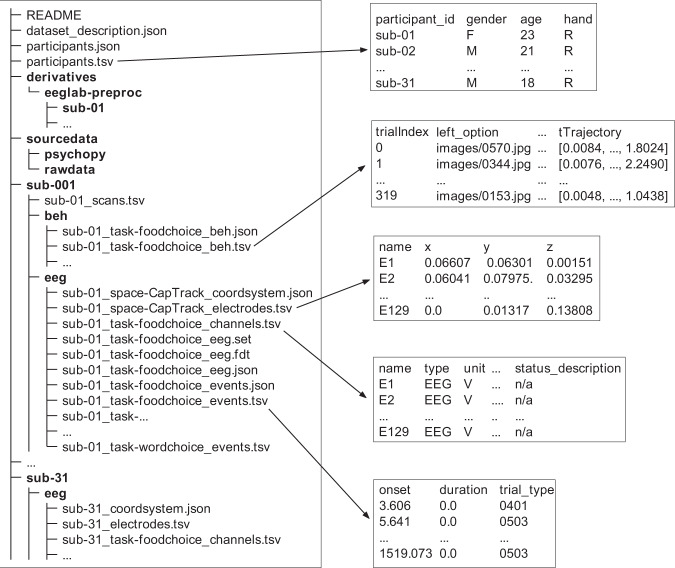
File structure of the repository. The left side shows the overview directory tree of our repository, and the arrows point to the content preview of corresponding files. The bold items represent folders.

The dataset can be accessed via the OpenNeuro link, organized following the EEG-BIDS^17^ specification, which was an extension to the brain imaging data structure for EEG. The overview directory tree of our dataset and part of the meta-data are shown in Fig. 2.

The repository contains three regular BIDS files, 31 participants’ data folders, a *sourcedata* folder, and a *derivatives* folder. The stand-alone files offer an overview of the dataset: (i) *dataset_description.json* is a JSON file depicting the dataset, such as the objective, acquisition time, and location; (ii) *participants.tsv* contains participants’ demographic information, such as age, sex, and handedness; (iii) *participants.json* describes the column attributes in *participants.tsv*. Each participant’s folder contains two folders named *beh* and *eeg* respectively and one file *sub-xx_scans.tsv*. The TSV file contains information about the scanning time of each task. The *beh* folder contains the corresponding behavioral data such as stimulus, response time, mouse trajectory, etc. The *eeg* folder contains the minimally processed raw EEG data, channels and marker events files of four sessions. The EEG data was converted from raw metafile format (*.mff* file) to EEGLAB^18^ dataset format (*.set* file) using the EEGLAB toolbox in MATLAB since EEG-BIDS is not officially compatible with the *.mff* format. All data was formatted to EEG-BIDS using the MNE-BIDS^19^ package in Python. The *sourcedata* folder contains two folders, *psychopy*, and *rawdata*, corresponding to the presentation scripts for all tasks and the raw EEG data in metafile format, respectively. Finally, the *derivatives* folder contains preprocessed EEG data, including resting-state and all three task sessions.

### EEG data preprocessing

**Fig. 3:**
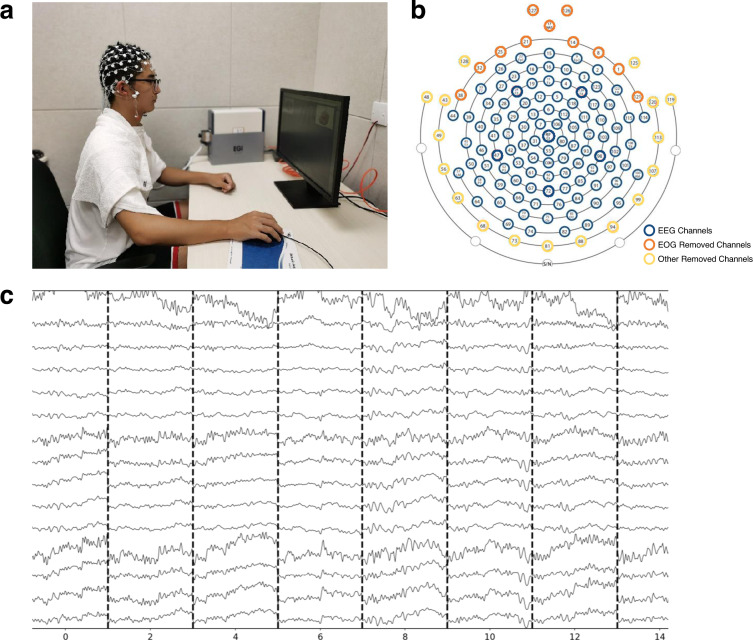
Data collection platform. (**a**) Data acquisition scenario with the computer mouse and recording EEG by a 128-channel EGI system. The participant has provided informed consent for the publication of the image. (**b**) The electrodes were kept or removed in the analyses. (**c**) Visualization of individual (*subject-09*) data after preprocessing.

For the data of binary choice tasks, standard preprocessing operations including resampling (100 Hz) and filtering (0.1–30 Hz) were performed in the EEGLAB. Moreover, 11 channels close to the left and right eyes were used as the EOG channels, and 17 channels with extensive artifacts were removed^20^ for further data processing and visualization, as shown in Fig. 3b. We removed bad channels with the *clean_rawdata* plugin and did spherical interpolation further. Then we converted data to average reference and rejected bad data periods with *clean_rawdata* again. Next, we ran ICA decomposition and used ICLabel^21^ for automatic independent component labeling and rejection. After preprocessing, we extracted data epochs from −200 ms to 800 ms at the stimulus onset with a mean baseline correction from −200 ms to the onset time. To provide an overview of the EEG signals, we presented an exemplar EEG result after preprocessing in Fig. 3c.

The resting-state EEG data preprocessing is basically identical with the task EEG except for minor parameter tuning for the subsequent microstate processing^22^. After standard procedures such as resampling, filtering, and artifacts removal on continuous data, we extracted the first 6-second data after fixation onset and segmented it into 2000 ms epochs, with −100 ms to 0 ms for baseline correction^8,23^.

### Behavioral and mouse-tracking preprocessing steps

**Fig. 4:**
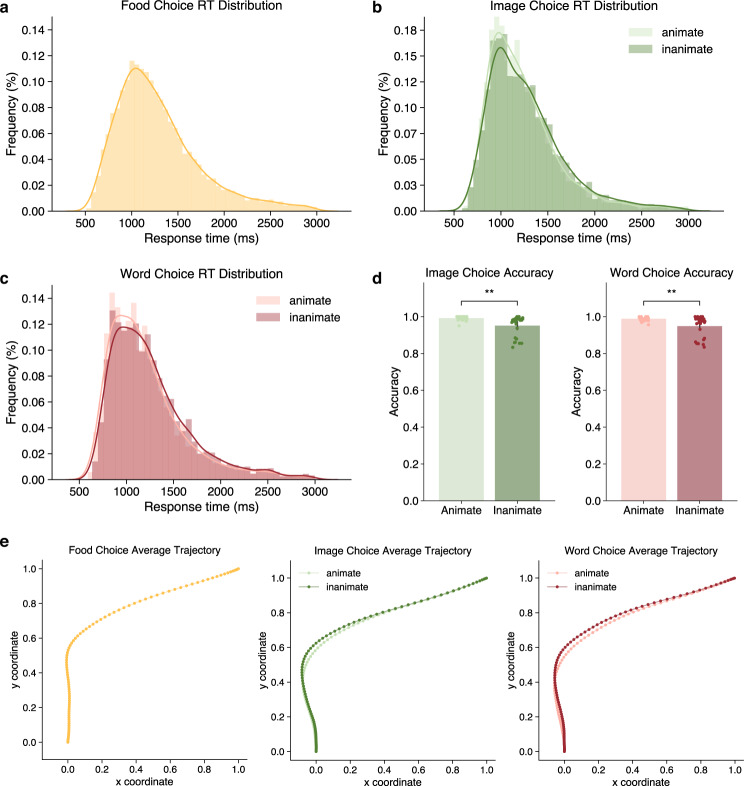
Behavioral results of the tasks. (**a**) Reaction time distribution of the food choice task. (**b**) Reaction time distribution of different semantic categories in the semantic category task with image modality. (**c**) Reaction time distribution of different semantic categories in the semantic category task with text modality. (**d**) Accuracy of different semantic categories in the semantic category tasks with image/text modality. (**e**) Average trajectory of mouse movements in three different tasks.

The behavioral analyses were performed separately, including reaction times (RT), accuracy, and mouse trajectory. RT and accuracy results were visualized in Fig. 4a–d respectively.

Mouse-tracking data was preprocessed temporally and spatially^24^. Temporally, the time windows combining trials and responses were sliced into 101 identical time bins using linear interpolation to average their length across multiple trials. Spatially, the trajectories of the mouse-tracking data were flipped to the same side and transformed into standard coordinate space (bottom left: [0, 0], top right:[1, 1]).

## Technical Validation

We could extract at least two kinds of valuable behavioral data for all binary choice tasks: i) behavioral data associated with choices, such as choices that participants made or their response time during this procedure, ii) mouse trajectory data when participants made a choice. Several novel analyses can be done in neuroscience or BCI by combining behavioral data, mouse trajectory, and EEG data. For example, since mouse trajectory and EEG are both time-series data, their fusion would allow us to explore the association between human behavior and brain activity in decision making dynamics^25^. For the image/text-based semantic judgment task, we can combine ERP analysis with EEG source reconstruction algorithms to explore the neural representation of objects in different modalities with HD-EEG data^26^. Actually, a recent study from HajiHosseini and Hutcherson has investigated the time course of food choices with EEG^27^. In addition, researchers can decode or predict mouse position based on EEG signals to get better performance when using BCI to control computer mouse cursor for people with motor disabilities.

Four participants were excluded from further validation. Participant *sub-28* performed poorly on both the word choice and image choice tasks, with an accuracy of less than 90%. There was 20 Hz EEG noise on participant *sub-01* and *sub-05*. Participant *sub-19* was excluded because of poor EEG epoch numbers (less than 200 trials for both word choice and image choice tasks) after automatic data rejection and epoching. Only trials with correct response were reserved for the word choice and image choice task in both behavioral and EEG data. There were 101 channels left, and the bad channel percentages over all three tasks (i.e., food choice task, word choice task, and image choice task) were 1.96% ± 1.28 (Mean ± SD), 2.56% ± 1.76, and 2.74% ± 1.85, respectively. The algorithm automatically determines the independent component number, which is around 100. The ICLabel removed components percentages for each task were 3.85% ± 1.8, 3.07% ± 1.49, and 3.31% ± 1.03, respectively. Since we only care about the correct response, the total trial number for different tasks is reduced to 310 to 320. After data rejection and epoching, 7.76% ± 4.43, 8.49% ± 4.21, and 10.02% ± 5.62 of epochs were dropped for each task.

### Behavioral validation

Here we presented some basic behavioral results to validate the dataset availability. Figure 4a–c showed the distribution of response time across trials of three binary choice tasks, respectively. The response time under animate condition (*M* = 1214.49 ms, *SD* = 224.31) was significantly shorter than inanimate condition (*M* = 1266.94 ms, *SD* = 222.86) in the word choice task, *t*(26) = −5.21, *p* < 0.001. The response time under animate condition (*M* = 1239.54 ms, *SD* = 210.73) was also significantly shorter than inanimate condition (*M* = 1284.78 ms, *SD* = 224.1) in the image choice task, *t*(26) = −4.06, *p* < 0.001. Figure 4d compared the response accuracy in different conditions of image and word choice tasks. The accuracy under animate condition (*M* = 0.989, *SD* = 0.011) was significantly higher than inanimate condition (*M* = 0.949, *SD* = 0.057) in the word choice task, *t*(26) = 3.57, *p* < 0.005. The accuracy under animate condition (*M* = 0.992, *SD* = 0.012) was also significantly higher than inanimate condition (*M* = 0.951, *SD* = 0.057) in the image choice task, *t*(26) = 3.60, *p* < 0.005.

We averaged all mouse trajectories in the food choice task and across two conditions (animate vs. inanimate) in the image choice and word choice tasks. The averaged mouse trajectories from three tasks were visualized in Fig. 4e. We observed a larger deviation for the inanimate condition in both image choice and word choice tasks, which are consistent with previous work.

### EEG validation

#### Resting-state EEG

After the preprocessing, we used the Microstate^28^ (https://www.thomaskoenig.ch/index.php/software/microstates-in-eeglab) plugin in EEGLAB to analyze the resting-state EEG data. We conducted the microstate analysis and identified four topography states (see Fig. 8). In light of the sample results, the topography states were stable, and the individual topography states were overlapped with four grand averaged topography states.

#### Food choice task EEG

**Fig. 5:**
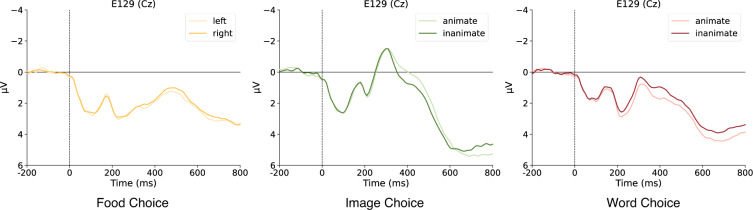
The comparison of the left vs. right choices during food task, and animate vs. inanimate comparison for image choice and word choice tasks over electrode Cz.

**Fig. 6:**
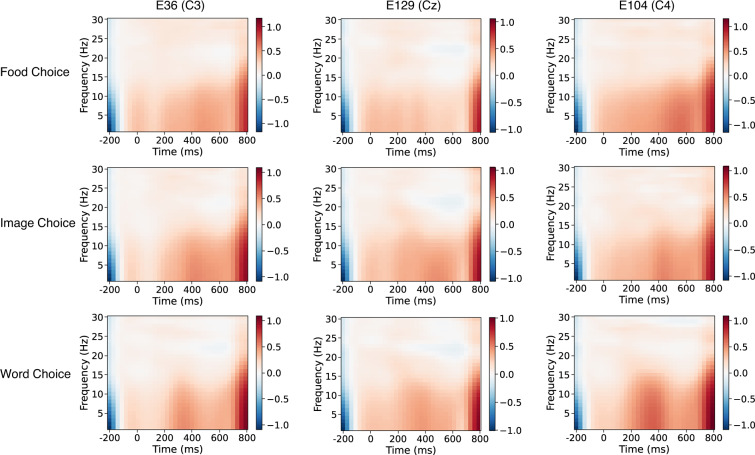
The group level averaged time-frequency maps of three tasks.

Following preprocessing, the EEG data of the food choice task was segmented in the time windows from −200 ms to 800 ms locked to food stimulus onset, concerning left vs. right choices. For each participant, the data were merged and averaged into two ERPs (left vs. right). The averaged ERPs were presented in Fig. 5. ERP amplitudes for the left and right choice over Cz demonstrated no significant difference. For the validation test, the spectral analysis was performed to measure ERP power from 1 to 30 Hz. The time-frequency analysis on the epoch data was conducted with the MNE-Python^29^ function of *tfr_morlet*. The time-frequency maps in Fig. 6 presented the global field power (GFP) results of subject 009 over C3, Cz, and C4. It identified higher alpha power in C3 and C4 than Cz due to the left and right motor responses. This was in line with a previous study which observed similar lateralized patterns in motor response, particularly for alpha power^30^.

#### Image choice task EEG

Likewise, the EEG data were segmented based on the image stimulus onset. The averaged ERPs and time-frequency results were displayed in Figs. 5 and 6. In particular, we observed more positive ERP amplitude over Cz for animate than inanimate stimuli. However, the averaged time-frequency maps across both conditions in Fig. 6 did not show a power difference between C3 and C4.

#### Word choice task EEG

Segmented EEG data were time-locked to word stimulus. Similarly, we displayed the averaged ERPs and time-frequency results in Figs. 5 and 6. For ERP amplitudes, we replicated more positive ERP amplitude over Cz for animate than inanimate words. This result is consistent with previous studies that showed larger negative N400 for inanimate word or sentence processing^31^. Again, the averaged time-frequency maps across both conditions in the word choice task did not show a power difference between C3 and C4.

Consistent with previous studies, we found longer RTs, larger deviations in mouse trajectories, and more negative conflict-related negativity for the inanimate condition than animate condition. Therefore, it validates the dataset from both behavioral and EEG features.

#### Decoding choices with EEG

**Fig. 7:**
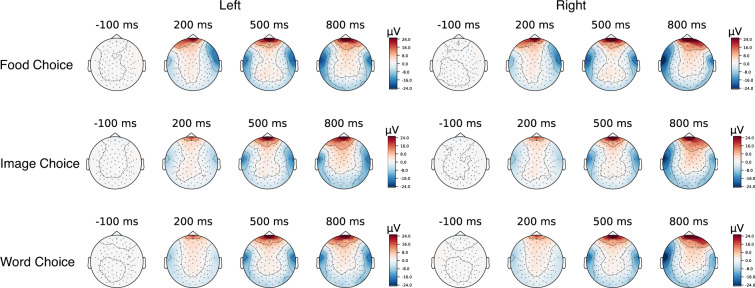
Averaged topographical distribution at −100 ms before the onset of the stimuli, 200 ms, 500 ms, and 800 ms after the stimulus onset during three tasks.

Decoding analyses were performed in MNE-Python combined with Scikit-learn, using the support vector machine classifier (with the function of *SVC*). For all three tasks, participants needed to click on the left or right option for a given pair of options. For simplicity, we decoded participants’ binary choices (left or right) for all three task-based EEG data. Here we extracted EEG epochs from −200 ms to 1800 ms since participants’ mean response time is over 1200 ms. The performance of classification is higher than the chance level (0.5). Specifically, the accuracy for the three tasks (i.e., food choice task, word choice task, and image choice task) were 0.67 ± 0.08, 0.73 ± 0.09, and 0.77 ± 0.1, respectively. The topomap for left or right choice in Fig. 7 also showed different patterns over time. The decoding results evaluated the potential of the data for future decoding use.

**Fig. 8:**
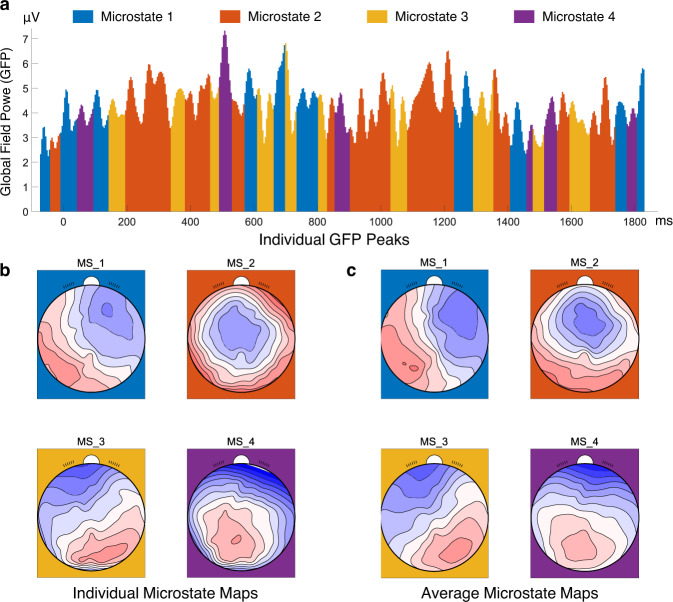
Microstate maps. (**a**) Individual (*subject-09*) global field power (GFP) peaks during 0 ms–2000 ms. (**b**) Individual (*subject-09*) microstate map. (**c**) Group level microstate map. *MS_1*: microstate 1, *MS_2*: microstate 2, *MS_3*: microstate 3, *MS_4*: microstate 4.

## Usage Notes

Most of the software or packages used for analyzing these data are freely available. All codes for preprocessing and plotting are openly accessed (see Section *Code availability*). We used a python package named squeak (https://github.com/eointravers/squeak) for the mouse trajectory data analysis of binary choice tasks. For task EEG data analysis, we used MNE-Python to generate formatted BIDS data, and plot ERP and time-frequency results based on the preprocessed data. Also, we used EEGLAB with plugins to preprocess all EEG data and do the microstate analysis for resting-state EEG data. For more detailed technical usage instructions, please refer to the GitHub repository.

There are three types of EEG event markers in all three binary choice tasks. The first is the trial index marker. Its value ranges from “0000” to “0319”, corresponding to the fixation starting from the first trial to the 320th trial, respectively. This type of marker mainly facilitates the correspondence between EEG data and behavioral data when the marker is missing.

The second is the stimulus marker, which appeared when the stimulus occurred and varied across tasks. There is only one stimulus marker in the food choice task, “0400”. In the image choice and word choice task, there are four types of stimulus markers, from “0400” to “0403”, which correspond to: (1) showing an animate object, the animate option appears on the left; (2) showing an animate object, the animate option appears on the right; (3) showing an inanimate object, the animate option appears on the left; (4) showing an inanimate object, the animate option appears on the right.

The third type is the response marker, which is locked to the option participants made. The food choice task has two response markers, “0500” and “0501”, denoting the left and right, respectively. In the image choice and word choice tasks, there are eight types of response marker, from “0500” to “0507”, which correspond to: (1) showing an animate object, the animate option appears on the left, left option selected; (2) showing an animate object, the animate option appears on the left, right option selected; (3) showing an animate object, the animate option appears on the right, left option selected; (4) showing an animate object, the animate option appears on the right, right option selected; (5) showing an inanimate object, the animate option appears on the left, left option selected; (6) showing an inanimate object, the animate option appears on the left, right option selected; (7) showing an inanimate object, the animate option appears on the right, left option selected; (8) showing an inanimate object, the animate option appears on the right, right option selected.

## Data Availability

The code used to preprocess the data and plot results is openly available on GitHub (https://github.com/andlab-um/MT-EEG-dataset). For more details about code usage, please refer to the GitHub repository.
