# Author Correction: A resource for assessing dynamic binary choices in the adult brain using EEG and mouse-tracking

**DOI:** 10.1038/s41597-022-01629-3

**Published:** 2022-08-19

**Authors:** Kun Chen, Ruien Wang, Jiamin Huang, Fei Gao, Zhen Yuan, Yanyan Qi, Haiyan Wu

**Affiliations:** 1 Centre for Cognitive and Brain Sciences, University of Macau, Taipa, Macau SAR China; 2 Department of Psychology, Faculty of Social Sciences, University of Macau, Taipa, Macau SAR China; 3 Department of Sociology, Faculty of Social Sciences, University of Macau, Taipa, Macau SAR China; 4 Faculty of Arts and Humanities, University of Macau, Taipa, Macau SAR China; 5 Faculty of Health Sciences, University of Macau, Taipa, Macau SAR China; 6 Department of Psychology, School of Education, Zhengzhou University, Zhengzhou, China

**Keywords:** Decision, Motor cortex

Correction to: *Scientific Data* 10.1038/s41597-022-01538-5, published online 16 July 2022

In this article the grant number CRG2021-00001-ICI relating to the University of Macau was omitted. The original article has been corrected.

